# Challenges in managing ruptured ectopic pregnancy complicated by hemorrhagic shock in an Ebola treatment unit in DR Congo: first reported case

**DOI:** 10.3389/fmed.2026.1848720

**Published:** 2026-08-21

**Authors:** Prince Imani-Musimwa, Emilie Grant, Daniel Mukadi-Bamuleka, Rigo Fraterne-Muhayangabo, Alfred Ombeni-Musimwa, Zacharie Tsongo-Kibendelwa, Olivier Nyakio-Ngeleza, Richard Kitenge-Omasumbu, Théophile Barhwamire-Kabesha, Dieudonné Sengey-Mushengezi-Amani, Placide Mbala-Kingebeni, Richard Bitwe-Mihanda, Juakali Sihali-Kyolov, Mija Ververs

**Affiliations:** 1 Department of Obstetrics and Gynecology, Faculty of Medicine and School of Public Health, University of Goma, Goma, Democratic Republic of Congo; 2 Centre de Recherche et d’Evaluation en Santé Globale et Action Humanitaire (CRESAH), Goma, Democratic Republic of Congo; 3 Faculty of Medicine and School of Public Health, Evangelical University in Africa, Bukavu, Democratic Republic of Congo; 4 Maternal and Infant Care Center, University of Washington Medical Center, Seattle, WA, United States; 5 Institut National de Recherche Bio-Médicale (INRB), Kinshasa, Democratic Republic of Congo; 6 Department of Medical Biology, School of Medicine, University of Kinshasa, Kinshasa, Democratic Republic of Congo; 7 Rodolphe Mérieux INRB-Goma Loaboratry, Goma, Democratic Republic of Congo; 8 Institut pour la recherche interdisciplinaire en sciences juridiques (JUR-I), Faculté de Droit et de Criminologie, Université Catholique de Louvain, Louvain-la-Neuve, Belgium; 9 Department of Internal Medicine, School of Medicine, University of Kisangani, Kisangani, Democratic Republic of Congo; 10 Ministère de la Santé Publique, Hygiène et Prevention, Kinshasa, Democratic Republic of Congo; 11 Department of Surgery, School of Medicine, Official University of Bukavu, Bukavu, Democratic Republic of Congo; 12 Department of Obstetrics and Gynecology, School of Medicine, University of Kinshasa, Kinshasa, Democratic Republic of Congo; 13 Department of Pediatrics and Neonatology, School of Medicine, University of Goma, Goma, Democratic Republic of Congo; 14 Department of Obstetrics and Gynecology, School of Medicine, University of Kisangani, Kisangani, Democratic Republic of Congo; 15 Center for Humanitarian Health, Johns Hopkins Bloomberg School of Public Health, Baltimore, MD, United States

**Keywords:** challenges in management, Ebola treatment unit, Ebola virus disease, exploratory laparotomy, hemorrhagic shock, RD Congo, ruptured ectopic pregnancy, Ebola survivor

## Abstract

**Background:**

Ectopic pregnancy (EP) is an obstetric emergency; early diagnosis is critical for maternal survival. In resource-limited settings, such as Ebola treatment units (ETU), delayed diagnosis or management of EP often leads to rupture and life-threatening hemorrhagic shock.

**Case presentation:**

We report the case of a pregnant woman in her 30s who was admitted to the ETU with suspected Ebola virus disease in the Democratic Republic of Congo (DRC). Her clinical presentation included fever and abdominal pain, which began 4 days before her admission. Thirteen months earlier, she had recovered from an Ebola infection. At admission, she presented with 8 weeks of amenorrhea and discrete pelvic pain. On vaginal examination, we noted a slightly enlarged uterus and palpated a small left adnexal mass. Laboratory exams confirmed malaria and pregnancy. Her EVD test was negative. Due to the absence of an ultrasound machine in the ETU, pelvic ultrasound was impossible. Supportive treatment was initiated. Nineteen hours after admission, the patient developed an acute surgical abdomen, with progressive left-sided pelvic pain, abdominal distension, and diffuse tenderness. A ruptured ectopic pregnancy was suspected and confirmed by diagnostic abdominal puncture, which revealed hemoperitoneum. She developed hemorrhagic shock. However, the ETU lacked an operating room, surgical equipment, and anesthetic drugs. Transfer to another ETU was not feasible due to the absence of a surgical-capable facility, while transfer to a community facility required at least a second negative EVD test 48 h after the first. We used an unused building that was part of the neighboring health center to perform an explorative laparotomy, which revealed an EP in the left tube with ampullary dislocation and active bleeding. We performed a left salpingectomy and homeostatic sutures. The real-time polymerase chain reaction (RT-PCR) tests for EVD performed at 48 and 72 h were negative. She had a normal postoperative course and was discharged from ETU to the community on the 14th day after surgery.

**Conclusion:**

Ruptured ectopic pregnancy (REP) remains a life-threatening obstetric emergency that may occur in ETUs, where resources and treatment conditions are limited. To protect maternal health, ETUs should ensure not only proper care for Ebola infection but also adequate capacity for the timely diagnosis and management of obstetric complications.

## 1 Background

Ectopic pregnancy (EP) is the implantation of the products of conception outside the uterine cavity. It accounts for 1–2% of naturally conceived pregnancies and 5% of pregnancies in women with a history of assisted reproductive technology (1). Several risk factors of EP have been described, including pelvic inflammation (2), which has also been reported among Ebola survivors (3). An estimated 8.3 million women worldwide experienced EP in 2021 (95% UI: 6.65–10.42) (4). Although the incidence and mortality have declined significantly in developed regions between 1990 and 2019, rates remain disproportionately high in low-income countries (5). EP is a life-threatening obstetric emergency with a high risk of maternal mortality. It is the leading cause of maternal death in the first trimester, historically accounting for 4–10% of early pregnancy deaths and approximately 6,400 deaths worldwide annually (6). Two studies from India and Ghana reported EP incidences of 0.98% (74/1521) and 0.78% (115/14,450), respectively. Among these cases, ruptured ectopic pregnancy (REP) accounted for 81.0 and 71.3% of EP cases, respectively, with the majority of patients admitted late in a state of hemorrhagic shock (7, 8). The management of REP is complex and involves appropriate diagnostic tools, surgical intervention, and well-trained staff. However, a majority of low- and middle-income countries lack resources required to successfully manage REP cases. Similar challenges have been reported in ETUs managing pregnant women with EVD in resource-limited settings (9–11), where maternal mortality remains extremely high. A recent systematic review and meta-analysis estimated a pooled maternal mortality of 67.8% (95% CI 49.8–83.7, *I*^2^ = 85%, *p* < 0.01) (12), highlighting the severe prognosis of EVD during pregnancy. Reported causes of death include severe viral hemorrhagic disease, multi-organ failure, shock, and obstetric complications such as miscarriage, placental abruption, preterm labor, and postpartum hemorrhage (12, 13), all of which are worsened by limited access to supportive care and delayed interventions in outbreak settings (11, 14, 15). Importantly, REP, a potentially fatal obstetric emergency, has not been documented as a cause of maternal death in ETU-based studies. This may reflect underdiagnosis and the focus on Ebola-related systemic complications, rather than its absence, and represents a critical knowledge gap. Limited diagnostic and therapeutic resources in ETUs make timely identification and management of obstetric emergencies extremely challenging (9, 14). These challenges are further amplified in pregnant women with suspected or confirmed EVD who have a concurrent EP, particularly when access to ultrasound, *β*-hCG measurement, medical therapy, or surgical intervention is limited, as illustrated by this case. Our patient narrowly survived a life-threatening REP complicated by hemorrhagic shock in a resource-limited Ebola outbreak setting. This case report aims to highlight the challenges of providing emergency obstetric care in ETUs and under resource-constrained conditions and to inform future clinical and public health preparedness for pregnant women in Ebola outbreaks. To our knowledge, this is the first documented case of REP in a patient admitted to an ETU.

## 2 Case presentation

During the 10th EVD outbreak, we admitted a woman on suspicion of EVD to the ETU in Eastern DRC. At the time of admission, she was in her 30s, weighed 68 kg, and was in her first trimester of pregnancy. Her clinical presentation included headaches, dizziness, fever, vomiting, abdominal pain and arthralgia, which began 4 days before her admission. She unsuccessfully self-medicated with paracetamol, ibuprofen and papaverine for 3 days before consulting the ETU.

Her medical history included EVD survival 13 months prior to the current episode of the illness. At that time, she had been exposed to a confirmed case, which motivated her to receive the Ervebo® vaccine, Recombinant Vesicular Stomatitis Virus-Ebola Zaire (rVSV-ZEBOV, Merck, Philadelphia, USA). However, 5 days following vaccination, she developed EVD symptoms, and 4 days after symptom onset, she was admitted to the ETU, where she tested positive for Ebola virus through real-time polymerase chain reaction (RT-PCR) using the GeneXpert® assay (Cepheid, Sunnyvale, CA, USA). She was treated with mAb114, Ebanga® (Ansuvimab, Ridgeback Biotherapeutics, USA), according to the PALM Study randomization protocol (16). She recovered and was discharged after 27 days of hospitalization in the ETU.

At her current readmission in the ETU, i.e., 13 months following her recovery from EVD, she was gravid 3, parity 1, with amenorrhea of 8 weeks and 2 days.

On clinical examination, she was alert and oriented and showed no signs of anemia or jaundice. Apart from a fever of 39.2 °C, her vital signs were normal (Table 1). Chest expansion was symmetrical and preserved. The abdomen was normotensive, with discrete pelvic pain. Fetal heart tones could not be assessed due to the unavailability of ultrasound equipment in the ETU. The vulva was clean. A speculum evaluation was not feasible. The ETU was not equipped with speculums or gynecological beds. On vaginal examination, the cervix was posterior, long, soft, and closed. The uterus was slightly enlarged, with a sensation of a small left adnexa mass, suggestive of a cyst, pedunculated myoma, or an EP. The Douglas was free, with a slight pelvic pain on cervical mobilization. Capillary refill time (CRT) was <3 s. The pregnancy rapid test was positive, and the malaria rapid diagnostic test (RDT) detected the *Plasmodium falciparum* strain. Blood glucose was 97 mg/dL, hemoglobin was 11 g/dL, platelet count was 287,000/mm^3^ and the blood group was O rhesus positive. The EVD test performed through RT-PCR was negative. We made a working diagnosis of an infectious syndrome (malaria and/or EVD relapse) complicated by a threat of abortion. A pelvic ultrasound could not be performed as we did not have access to a machine. She was treated with artesunate 2 × 140 mg/day/5 days, paracetamol 2 × 1 g/day, ceftriaxone 2 g/day/7 days, albendazole 1 × 400 mg, multivitamins 3 × 1 tab/day/14 days, tramadol 2 × 100 mg/day, omeprazole 2 × 40 mg/day/10 days, papaverine 2 × 40 mg/day, folic acid 1 × 5 mg/day/30 days, and oral rehydration solution (17).

**Table 1:** Chronological clinical course, interventions, and outcomes.

Time	Symptoms	Vital signs	Diagnostic hypotheses	Laboratory results	Interventions	Outcomes/evolution
0 h (Admission)	Headache, dizziness, fever, vomiting, abdominal pain, arthralgia	T° 39.2 °C, BP 122/74 mmHg, HR 86 bpm/min, RR 20/min, SaO₂ 98%	Suspected infectious syndrome (malaria and/or EVD), threatened abortion with adnexal mass (ovarian cyst, fibroid, or EP)	Hb 11 g/dL, Plt 287 × 10^9^/L, Glucose 97 mg/dL, Malaria RDT+, Pregnancy test+, EVD RT-PCR−	Artesunate, ceftriaxone, paracetamol, tramadol, Albendazole, omeprazole, papaverine, folic acid, multivitamins, ORS, IV fluids	Initial stabilization; slight improvement
10 h	Progressive accentuation of pelvic pain, nausea, light amount of vaginal bleeding	T° 38.5 °C, BP 111/70 mmHg, HR 105 bpm, RR 24/min, SaO₂ 96%	First-trimester hemorrhage/threatened abortion, suspected EVD relapse	Hb 10 g/dL, Plt 265 × 10^9^/L, Malaria RDT + Glucose 124 mg/dL	IV fluids, haemaccel, phloroglucinol	Mild symptom improvement in symptoms
19 h	Sharp left pelvic pain, bright red vaginal bleeding, agitation, abdominal bloating	T° 38.2 °C, BP 85/60 mmHg, HR 122 bpm/min, RR 26/min, SaO₂ 94%	Suspected REP complicated by hemorrhagic shock	Hb 8 g/dL, Plt 152 × 10^9^/L, Malaria RDT + Glucose 82 mg/dL	Oxygen 5 L/min, IV fluids, blood transfusion, Diagnostic abdominal puncture	Deterioration; shock onset
22 h	Increasing pain, ongoing bleeding, pallor, cool extremities, CRT > 6 s	T° 39.4 °C, BP 70/40 mmHg, HR 138 bpm, RR 32/min, SaO₂ 84%	Hemorrhagic shock secondary to REP	Hb 6 g/dL, Plt 112 × 10^9^/L, Glucose 61 mg/dL, Malaria RDT+	IV fluids, multiple blood transfusions, vasopressors, oxygen	Further deterioration; life-threatening
30 h	Severe generalized abdominal pain, tenderness, fever	T° 38 °C, BP 60/34 mmHg, HR 156 bpm, RR 36/min, SaO₂ 82%	Confirmed REP	Hb 5 g/dL, Plt 104 × 10^9^/L, Glucose 143 mg/dL, Malaria RDT+	Exploratory laparotomy, left salpingectomy, drain placement	Slight postoperative hemodynamic stabilization
48 h	Mild, localized peri-incisional pain	BP 102/64 mmHg, HR 112 bpm, RR 24/min, Temp 37.5 °C, SaO₂ 97%	Post-operative recovery	Hb 6.9 g/dL, Plt 126 × 10^9^/L, Glucose 126 mg/dL, Malaria RDT+, RT-PCR−	Surveillance	Hemodynamic stabilization postoperatively
72 h (day 2 post-op)	Mild, localized peri-incisional pain	BP 110/69 mmHg, HR 98 bpm, RR 22/min, Temp 37.2 °C, SaO₂ 98%	Postoperative recovery	Hb 7.6 g/dL, Plt 131 × 10^9^/L, Glucose 76 mg/dL, Malaria RDT−, RT-PCR−	Surveillance	Patient stabilized
336 h (day 14 Post-op)	Asymptomatic	Normal	Full recovery	Normal labs	Discharge from ETU, Contraceptive implant insertion	Complete clinical recovery

Approximately 10 h after her admission the same day, despite the treatment initiated, she experienced progressive accentuation of pelvic pain, nausea, and a small amount of vaginal bleeding. She was febrile, normotensive, tachycardic, and tachypneic with SaO_2_ at 96% on air. Pelvic tenderness was more pronounced on the left flank, and her vulva was stained with a small amount of coagulated blood; vaginal exam revealed a slight sensitivity of the Douglas. The CRT was approximately 4 s, hemoglobin was 10 g/dL, and platelet count was 265,000/mm^3^. A diagnosis of first trimester hemorrhage complicated by a threatened abortion in a suspected EVD patient was established. We were unable to perform an ultrasound or a quantitative beta chorionic gonadotropin hormone (HCG) test in the ETU because the required equipment was not available. Intravenous Haemaccel^®^ (Polygeline, Piramal Critical Care Ltd., India) 1,000 mL and Phloroglucinol^®^ (2 × 80 mg) were added to the treatment, and a slight decrease in symptoms was observed.

**Figure 1:**
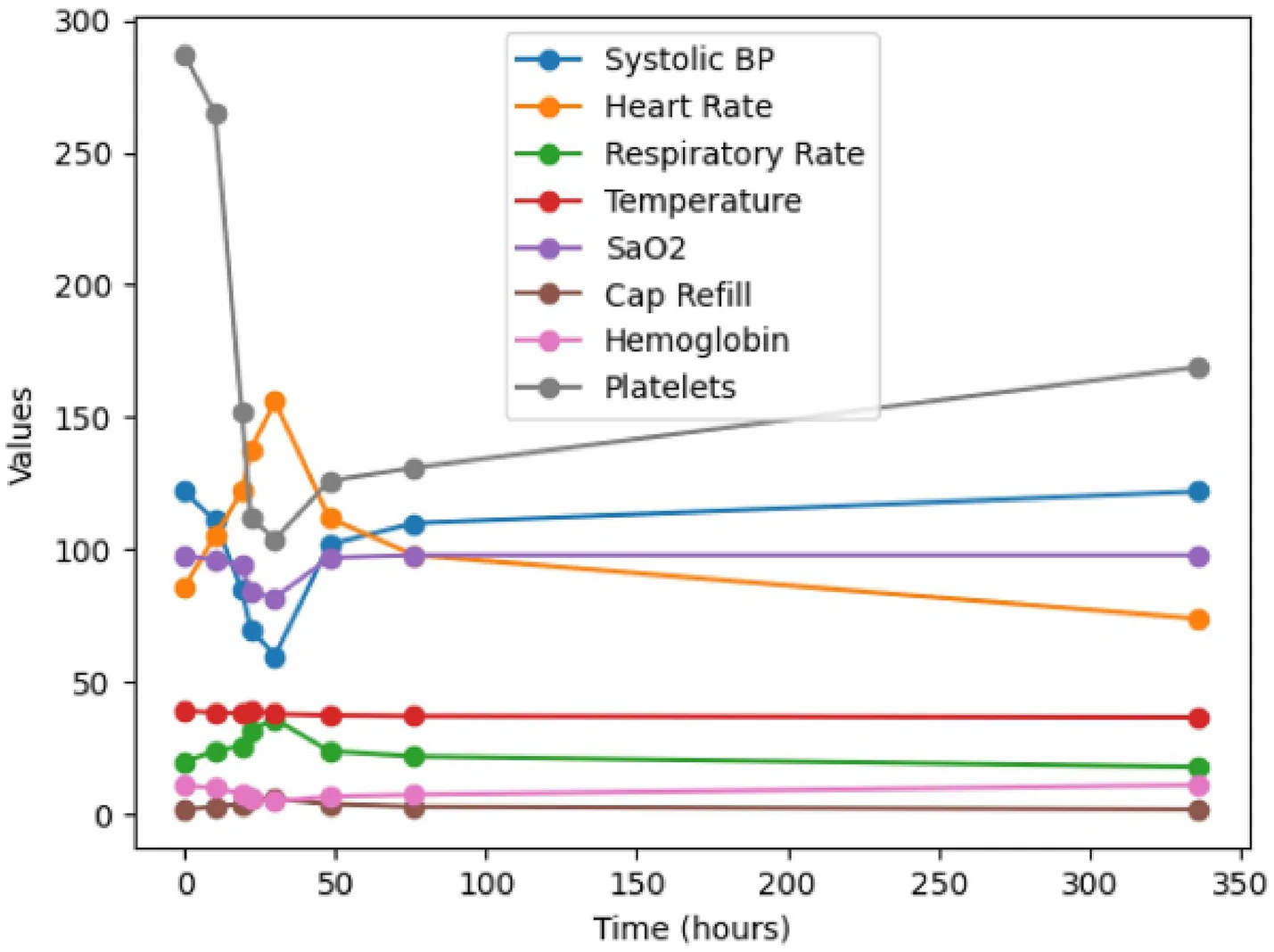
Trends in vital signs and hematologic parameters over time.

Nineteen hours after admission, the patient developed hemorrhagic shock. She experienced sharp, stabbing left pelvic pain followed by a small amount of bright red vaginal bleeding 15 min later. Her general condition was marked by restlessness. Her clinical evaluation revealed pink colored buccal and conjunctival mucosa, fever, hypotension, tachycardia, tachypnea, and mild hypoxemia in air (Table 1, Figure 1). We introduced oxygen therapy at 5 L/min and continued with 900 mL of blood transfusion and mixed infusion fluids.

Progressive abdominal bloating was noted with generalized tenderness, moderate vaginal bleeding, a closed cervix, and bulging and painful Douglas vacuum. We noted cutaneous and mucosal pallor and cool extremities with a CRT of > 6 s, hemoglobin of 8 g/dL, and a platelet count of 152,000/mm^3^. We suspected REP. Despite the prohibition of invasive procedures in the ETU (9), the acute surgical abdomen of our patient required that we perform, in strict compliance with Infection Prevention and Control (IPC) measures, a diagnostic abdominal puncture that revealed hemoperitoneum. We confirmed REP complicated by hemorrhagic shock in a patient suspected of an EVD infection. In the absence of a surgical-capable ETU and amid security and logistical constraints, transfer to another facility was not feasible. Furthermore, according to protocol, the patient could not be referred to a community health facility for surgery until a second and/or third negative EVD PCR test performed 48–72 h later (18) was obtained. At that time, only 22 h had elapsed since the first negative EVD PCR test.

Despite resuscitation measures, the patient’s condition continued to worsen, with further abnormalities in vital signs despite supportive care, including oxygen therapy. Her malaria test was still positive, her hemoglobin was 6 g/dL, and the platelet count was 112,400 mm^3^ (Table 1, Figure 1). We faced the dilemma of breaking protocol and potentially exposing others in the receiving health center to a hemorrhaging EVD patient in the absence of the second RT-PCR results or continuing to observe her deteriorating state without the option of further intervention in the ETU.

The ETU was situated on the same compound as a health center, which divided its services into several separate buildings. According to protocol, a physical barrier was constructed to separate the ETU from the health center buildings. We moved the physical barrier to annex the building in closest proximity to the ETU, which we then transformed into an operating room and where we performed surgery using the limited equipment available to us.

Thirty hours from admission, the blood pressure (BP) was 60/34 mm Hg, heart rate (HR) was 156 beats/min, respiratory rate (RR) was 36 breaths/min, temperature was 38.3 °C, and oxygen saturation (SaO_2_) was 82% under oxygen therapy, with Hb 5 g/dL and platelet count (PLT) of 104 × 10^9^/L. With the health center’s minimal surgical kit allocated, under full PPE, we performed an explorative laparotomy under general anesthesia. We found a massive hemoperitoneum of approximately 3 L of blood, with REP in the left tube and ampullary dislocation. The patient was actively bleeding, with multiple visceral-tubular and parietal adhesions. We performed a left salpingectomy, and homeostatic sutures were performed. The contralateral adnexa was macroscopically normal. The abdominal cavity was irrigated and toileted with normal saline (0.9% sodium chloride). An evacuation probe was inserted into the abdominal cavity, followed by the suture of the abdominal wall. The room and materials were decontaminated in compliance with the IPC protocols related to the Ebola context (17).

In total, the patient received 3,450 mL of blood, replacement fluids, calcium gluconate, vasopressors, ceftriaxone and metronidazole, tramadol, intravenous paracetamol, and oxygen therapy for 2 days after her surgery. Nearly 48 h after the first negative EVD test, i.e., 16 h after surgery, the second RT-PCR test was negative. Given her persisting symptoms, we conducted a third test at 72 h with negative results; she was then declared EVD negative. She remained hospitalized in the same building in which we had performed the surgery. She had a normal postoperative course with favorable clinical and laboratory evolution and was discharged from the ETU to the community on the 14th day after surgery. After recovery, she received a contraceptive implant, which was removed 1 year later. She subsequently conceived an intrauterine pregnancy with normal evolution and gave birth to a healthy female newborn without apparent malformations.

## 3 Discussion

Our patient, a survivor of EVD, presented 13 months later to an ETU with suspected Ebola infection during pregnancy. During her hospitalization, she developed REP complicated by hemorrhagic shock, likely related to delayed diagnosis in a highly constrained outbreak setting. This case illustrates the complex management of surgical obstetric emergencies in a pregnant woman admitted to an ETU, highlighting the diagnostic and operational challenges inherent to outbreak settings, where access to imaging, laboratory confirmation, and surgical resources is often limited. Beyond its clinical severity, this case underscores the need for adaptive clinical decision-making and system-level preparedness to ensure timely and safe obstetric care during high-risk infectious disease outbreaks. In addition to these operational constraints, it also raises important questions regarding potential long-term reproductive sequelae among Ebola survivors.

Although several factors are associated with EP (2), in our case, the cause of EP remains uncertain. To date, there is no established clinical or histopathological evidence demonstrating that Ebola infection causes pelvic inflammatory disease, tubal scarring, or ciliary dysfunction predisposing to EP in humans. However, Ebola virus has been shown in non-human primate models to infect reproductive organs, including ovarian and uterine tissues (19), eliciting inflammatory responses that could theoretically have subclinical effects on tubal mucosa or ciliary function. This represents an important knowledge gap, and further targeted reproductive and histopathological studies among female Ebola survivors are warranted.

A recent systematic review reported that approximately 14% of female survivors of EVD experience reproductive health sequelae, including pelvic inflammatory disease (3). In our case, there was no history of pelvic inflammatory disease prior to Ebola infection. The EP may have resulted from a post-inflammatory adhesion syndrome observed intraoperatively, plausibly caused by these post-EVD inflammatory complications 13 months after infection, as pelvic inflammatory disease is a well-established cause of tubal damage.

Despite confirmation of an acute surgical abdomen and the lack of surgical capacity in the ETU, persistent EVD symptoms prevented transfer to a community facility without at least a second negative test. Some studies have reported false-negative Ebola RT-PCR results of tests performed on admission and within 48 h, leading to discharge from the ETU of infected and contagious individuals. To circumvent this concern, it is important to perform at least two other tests at 48 and 72 h (20, 21), particularly when EVD symptoms persist, as was observed in our case. The onset of EVD symptoms in survivors should be thoroughly investigated and appropriately managed. After EVD recovery, Ebola RNA can persist in immune-privileged sites, such as cerebrospinal fluid, semen, breast milk, vaginal secretions, and the plasma-like aqueous humor, for months or years and can provoke relapse and/or sexual transmission (22–24). In this case, transfer out of the ETU was very dangerous due to persistent EVD symptoms. Due to these factors, three tests were performed according to protocol. Her third test 72 h after admission confirmed her negative EVD status. Before this third test, we hypothesized an Ebola infection (relapse or re-infection) because of the persistence of symptoms. The risk of reinfection or reactivation of Ebola virus increases progressively in survivors who have been treated with monoclonal antibodies because of the rapid decrease in anti-Ebola antibody production that they induce in the period following recovery (25). In addition, despite having recovered from Ebola infection for 13 months and given the gradual decrease in the production of anti-Ebola antibodies as previously mentioned, the risk of re-infection was also possible, especially as the epidemic was still ongoing.

According to the literature, REP is the leading cause of maternal mortality in the first trimester of gestation. Mortality is primarily related to delayed diagnosis and, consequently, the lack of appropriate management (1). In our case, various risk factors compounded: Immune vulnerability induced by pregnancy, hemorrhagic shock due to REP, and malaria infection threatened our patient’s survival. Malaria remains one of the most common co-infections among patients admitted to the ETU. During the West Africa Ebola outbreak, one study conducted in Sierra Leone revealed that malaria co-infection was associated with 72% of EVD deaths and specifically with 25% of maternal mortality in the ETU (26).

Although this may be the first case of REP reported in an ETU, we cannot assume that it is the first to occur in a suspected and/or confirmed Ebola patient; the conditions for early diagnosis of this obstetrical emergency remain limited. Considering the symptoms of patients admitted to ETUs and their risk of both septic and hemorrhagic shock, the assessment of vaginal bleeding alone is insufficient to guide targeted interventions. Undetected REP can lead to hemorrhagic shock masked by EVD symptoms. In this context, the patient’s survival depends on early management, which, in turn, depends on accurate early diagnosis. These criteria are only possible with adequate technical resources, which we did not have in the ETU.

Despite the occurrence of vaginal bleeding in some REP cases, the rupture is often misdiagnosed as abortion, a common EVD symptom. In such situations, treatment includes tocolysis of the pregnancy or attempted evacuation of an empty uterus.

Missed early diagnosis of REP leads to misguided management, which is dangerous for the patient and may negatively impact maternal outcome. We used basic clinical resources to diagnose REP in the first trimester of pregnancy. Although REP was suspected 19 h after admission, laparotomy was performed 11 h later. During this period, the patient showed progressive hemodynamic deterioration before definitive surgical management. This delay may be attributed to diagnostic uncertainty due to overlapping symptoms with malaria and EVD and limited surgical capacity.

This case presents a clinical emergency in a pregnant woman admitted to the ETUs whose diagnosis and management were complicated by the limited access to diagnostic tools. Considering the context of our interventions, her survival is exceptional. Despite her recovery, this study highlights insufficiencies for pregnant women treated in ETUs.

In this case, the proximity of the ETU to a slightly better-equipped health center was of vital importance in saving the patient’s life. However, to improve maternal care during EVD outbreaks, we recommend that outbreak response planning consider the proximity of health centers and ensure the establishment of ETUs equipped with surgical instruments and operating rooms to manage gynecological and obstetric emergencies in both suspected and confirmed cases, while fully complying with IPC standards.

Beyond the clinical relevance of this case, several important outbreak-related limitations should be acknowledged, including the inability to obtain preoperative imaging of EP due to the lack of an ultrasound machine in the ETU, as well as the absence of intraoperative images and histopathological confirmation. These limitations were precluded by strict infection prevention and control protocols. Surgery was performed in an Ebola Red zone, where the use of photographic or imaging devices is prohibited, as mandatory post-use decontamination with a 0.5% chlorine solution irreversibly damages electronic equipment. Similarly, trophoblastic tissue sampling for histopathological examination was not feasible, as Ebola protocols prohibit the transfer of potentially infectious biological tissues to external laboratories due to the risk of community exposure. These limitations reflect systemic safety requirements rather than diagnostic uncertainty and should be interpreted within the context of emergency care delivery during high-risk infectious disease outbreaks.

## 4 Conclusion

Obstetric emergencies occur in ETUs, where limited technical capacity and clinical resources may compromise maternal and fetal outcomes. Early recognition and management, supported by functional and well-equipped operating rooms and trained providers, are essential for safe care. Obstetric procedures, including uterine evacuation and vaginal deliveries, have been successfully performed in ETUs, demonstrating that, with proper planning, infrastructure, strict adherence to IPC measures, and well-trained staff, obstetric surgical emergencies can be safely managed in these high-risk settings. Strengthening ETU capacity is critical to reducing maternal and neonatal morbidity and mortality and integrating obstetric emergency care into outbreak preparedness and response plans globally.

## Data Availability

The raw data supporting the conclusions of this article will be made available by the authors, without undue reservation.
